# Role of pretreatment variables on plasma HIV RNA value at the sixth month of antiretroviral therapy including all first line drugs in HIV naïve patients: A path analysis approach

**DOI:** 10.1371/journal.pone.0213160

**Published:** 2019-03-11

**Authors:** Carlo Mengoli, Monica Basso, Samantha Andreis, Renzo Scaggiante, Mario Cruciani, Roberto Ferretto, Sandro Panese, Vinicio Manfrin, Daniela Francisci, Elisabetta Schiaroli, Gaetano Maffongelli, Loredana Sarmati, Massimo Andreoni, Franco Baldelli, Giorgio Palu', Saverio Giuseppe Parisi

**Affiliations:** 1 Department of Molecular Medicine, University of Padova, Padova, Italy; 2 Center of Diffusive Diseases, ULSS 20, Verona, Italy; 3 Clinical Infectious Diseases, Santorso Hospital, Santorso (VI), Italy; 4 Clinical Infectious Diseases, Mestre Hospital, Venezia, Italy; 5 Clinical Infectious Diseases, Vicenza Hospital, Vicenza, Italy; 6 Clinical Infectious Diseases, University of Perugia, Perugia, Italy; 7 Clinical Infectious Diseases, Tor Vergata University, Roma, Italy; University of Pittsburgh, UNITED STATES

## Abstract

**Background and aims:**

We investigated the conditioning roles of viral tropism and other variables on plasma HIV RNA levels after 6 months of combination antiretroviral therapy (cART) in an HIV-infected Italian naïve population using regression tree, random forest regression, and path analysis (PA). Patients in this multicenter observational study were treated with all antiviral drugs that are currently recommended as first-line therapies.

**Methods:**

Adult patients with chronic HIV infection were enrolled at the beginning of first-line cART (T0). The main variables were age, gender, tropism, “lcd4_0” and “lcd4_6” (log_10_ CD4+counts at T0 and after 6 months of cART, respectively), and “lrna0” (log_10_ HIV RNA at T0). Regression tree and random forest analyses were applied. The predictive effect on lrna6 (log_10_-transformed plasma HIV RNA after 6 months of cART) was also investigated via PA (x4->lcd4_0->lrna0->lrna6) with a treatment selection step included as a dependent (mediator) variable for each third drug and, as predictive covariates, age, female, x4_10, x4_5, lcd4_0, and lrna0. Tropism was assessed in plasma using the Geno2pheno algorithm with 2 false positive rate (FPR) cut-offs: 5% (x4_5) and 10% (x4_10).

**Results:**

The study included 571 subjects (21% x4_10 and 10.7% x4_5). The only important predictor of lrna6 was lrna0, and a positive indirect effect of bearing X4 virus in plasma was suggested. A significant direct positive effect of protease inhibitors on lrna6 was found (*p* = 0.022), and a significant negative effect of integrase strand transfer inhibitor (INSTI) was also detected (*p* = 0.003 for FPR ≤ 5% and *p* = 0.01 for FPR < 10%). PA predicted mean residual viremias of 40 copies/mL without INSTI and 3 copies/mL with INSTI.

**Conclusions:**

PA indicated a possible indirect role of HIV tropism on lrna6 with both FPR < 10% and ≤ 5%. Patients treated with INSTI had a predicted residual viremia of 3 copies/mL.

## Introduction

The most recent guidelines recommend the immediate administration of combination antiretroviral therapy (cART) irrespective of the CD4+ cell count in adult patients [1,2]. Multiple drugs with comparable efficacies are available for first line therapy in high-income countries, and most naïve HIV-1-infected patients achieve viral suppression after 6 months of therapy [2]. However, the pretreatment clinical and virological characteristics of patients, such as HIV RNA levels, influence the virological response [3]. The negative predictive value of pretreatment tropism (defined as harboring an X4 virus) on HIV RNA at week 24 in naïve patients receiving first-line antiretroviral therapy was described with two different study designs and statistical approaches by Seclen et al. [4] and by our group [5]. In the former, the authors performed a study on 428 patients included in the ArTEN study, which was a prospective randomized trial comparing the efficacies of nevirapine (nvp) versus atazanavir-ritonavir (atv/r), both in combination with fixed-dose tenofovir and emtricitabine. They demonstrated that being infected with an X4 virus was an independent negative predictor using linear and logistic regression models. Conversely, our work [5] included 262 patients who were treated at the discretion of the treating physician in a clinical practice setting with either abacavir/lamivudine or tenofovir disoproxil fumarate/emtricitabine as the backbone (BB) plus a protease inhibitor (pi) boosted by ritonavir (atv/r or darunavir (drv)/r or lopinavir/r) or a non-nucleoside reverse transcriptase inhibitor (efavirenz (efv) or nvp). Next, we built a path analysis model to explain the direct and mediated effects of the variables (which included tropism) on the final outcome and demonstrated a significant positive indirect effect of bearing the X4 virus on HIV RNA at the 6^th^month of therapy. Interestingly, a different false positive rate (FPR) was applied in the 2 studies (5.75% and 10%, respectively; both were interpreted using Geno2pheno) and no patient was treated with integrase strand transfer inhibitor (INSTI). Currently, INSTIs are the class of drug that has a leading role in HIV treatment in both naïve and experienced patients because of their efficacy, tolerability, and safety [6–8]. Raltegravir (ral) and elvitegravir (evg) belong to the first generation of INSTIs, and dolutegravir (dtg) is the only second-generation INSTI approved by the U.S. Food and Drug Administration (FDA) and is currently also used in Europe [2,9,10]. Few data are available about the role of tropism in patients treated with INSTIs. Armenia et al. [3] included 32 patients treated with ral in a multicenter study that demonstrated that an FPR ≤ 2% was associated with a lower rate of virological suppression in naïve subjects who were treated with different first-line cARTs, but the specific roles of the different drug combinations were not addressed.

The objective of this study was to investigate the conditioning roles of tropism (evaluated with FPR of both 5% and 10%) and the other main clinical and virological variables using regression tree analysis, random forest regression, and path analysis (PA) on the virological response after 6 months of cART in an HIV-infected Italian naïve population. Additionally, we sought to investigate the therapeutic activities of all antiviral drugs that are currently recommended as first-line therapies, including INSTIs. The population was treated in a multicenter observational context.

## Materials and methods

### Study design

Adult patients with a diagnosis of chronic infection with subtype B HIV-1 were consecutively included in the study from January 1, 2014, to April 30, 2016, at the begin of first-line cART in 6 Italian infectious disease centers, located in Padova, Santorso (Vicenza province), Vicenza, Venice, Perugia, and Roma. The decision to begin antiviral treatment and the choice of HIV drug combination followed the guidelines that were current at the initiation of the study. The physicians were unaware of the tropism of the plasma HIV strains of the patients.

All data were fully anonymized before they were accessed by the study authors. The patients provided informed written consent for the procedures and for the use of their blinded data for scientific evaluation. This study was conducted in accordance with the Declaration of Helsinki, and was approved by the Ethics Committee of Padova University Hospital (prot. 2606-12P).

Abacavir/lamivudine (abclam) or tenofovir disoproxil fumarate/emtricitabine (tdfftc) were used as BB, plus a third component: efv, nvp, rilpivirine (rpv), atv/r, drv/r, ral, dtg, or evg. Only patients who did not interrupt treatment or require treatment modifications due to intolerance and those with self-reported adherence > 95% were included in the study. Plasma levels of HIV RNA were assessed with the same commercial method in each of the six infectious diseases centers throughout the study period. The viro-immunological parameters were evaluated at 2 study points: T0 (before cART) and T6 (6^th^ months of cART). All patients had tropism tested at T0. Tubes containing EDTA were used to collect blood samples; plasma and cells were separated by centrifugation. Aliquots of plasma were stored at -80°C until the tropism analysis.

### Genotypic prediction of viral tropism

The genotypic analyses of the viral tropisms were performed on the plasma samples as previously reported [11]; we then applied quality controls of the results obtained as previously described in detail [5].

The bioinformatic tool Geno2pheno was used to interpret the generated V3 sequences with FPRs of 10% and 5% [12,13]. Geno2pheno is available at http://coreceptor.bioinf.mpiinf.mpg.de [14].

Useful amplification and sequencing of the V3 region was obtained in all plasma available from the subjects, so all subjects were analysed.

### Statistical analysis

The main variables were age (years), gender (binary, male versus female), tropism (binary, CCR5 versus CXCR4), “lcd4_0” (log_10_-transformed CD4+ cell count at T0), “lcd4_6” (log_10-_transformed CD4+ cell count after 6 months of cART), “lrna0” (log_10_-transformed plasma HIV RNA at T0), and “lrna6” (log_10_-transformed plasma HIV RNA after 6 months of cART). The variables gender and tropism were defined as being female and as being x4_10 (FPR < 10%) or x4_5 (FPR ≤ 5%), respectively in the analysis.

The virological effects of cART were evaluated using lrna6 as the outcome variable. The drugs used for cART were coded as binary variables. Binary variables were also created to evaluate the drugs as single agents or as classes. The classes were defined as follows: “nnx” (efv and nvp), “nn” (efv, nvp, and rlp), PI, and INSTI.

The mean values of a group of demographic, immunological, and virological (clinical, as in clinical interest) variables were calculated as the distribution of lrna0 (the virological predictor) and lrna6 (the virological outcome indicator). Conventional descriptive statistics were applied when appropriate.

#### Classifiers: Regression tree and random forest analyses

The capability to predict the main outcome (lrna6) by clinical variables (i.e., lrna0, lcd4_0, x4_10, x4_5 age, and gender) and the therapeutic drugs binary indicators (i.e., abclam, tdfftc, and the third drugs) was evaluated by regression tree analysis using the recursive partitioning approach.

The same variables were evaluated with random forest regression. The relevant software programs used were the “rpart” and “randomForest” packages of R version 3.3.2.

The endogenous treatment effect was estimated with a control-function regression adjustment as implemented with the Stata 14.2 “eteffects” module. With this method it was possible to check for the persisting endogeneity of the treatment due to unobservable variables. All 8 third drugs were submitted to evaluation, controlling for age, female, x4_10, x4_5, lcd4_0, lrna0, abclam, and tdfftc in the treatment selection model, and for age, female, x4_10, x4_5, lcd4_0, and lrna0 in the outcome model.

The same analyses were performed with the drug classes.

#### Path analysis

The predictive power on the main outcome (lrna6) by clinical variables (lrna0, lcd4_0, x4_10, x4_5, age, and gender) and each one of the third drug group (efv, nvp, atv/r, rpv, drv/r, ral, dtg, and evg) was also investigated by means of path analysis, using the SEM module of Stata v. 14.2. The model was based on the model previously published (5) putting forward the path: x4- > lcd4_0- > lrna0- > lrna6, with the addition of a treatment selection step includingas a dependent (mediator) variable each individual third drug, and, as predictive covariates, age, female, x4_10, x4_5, lcd4_0, and lrna0. Moreover, age and female were enclosed as exogenous predictors on each regression step. The estimation method was asymptotic distribution-free. The coefficients were standardized. The objective was to evaluate in an unbiased way the effect of various drugs in a context of an observational study, where the selection bias was clearly present. The model formulated aimed at closing the “backdoor” due to confounders conveying potential selection bias on the apparent treatment effect. The same model was applied once for each third drug, and once for each third drug class. Then, the model was used to obtain a prediction concerning the HIV plasma load (the dependent variable) under the two opposite conditions integrase inhibitor used versus integrase inhibitor not used.

## Results

**Fig 1:**
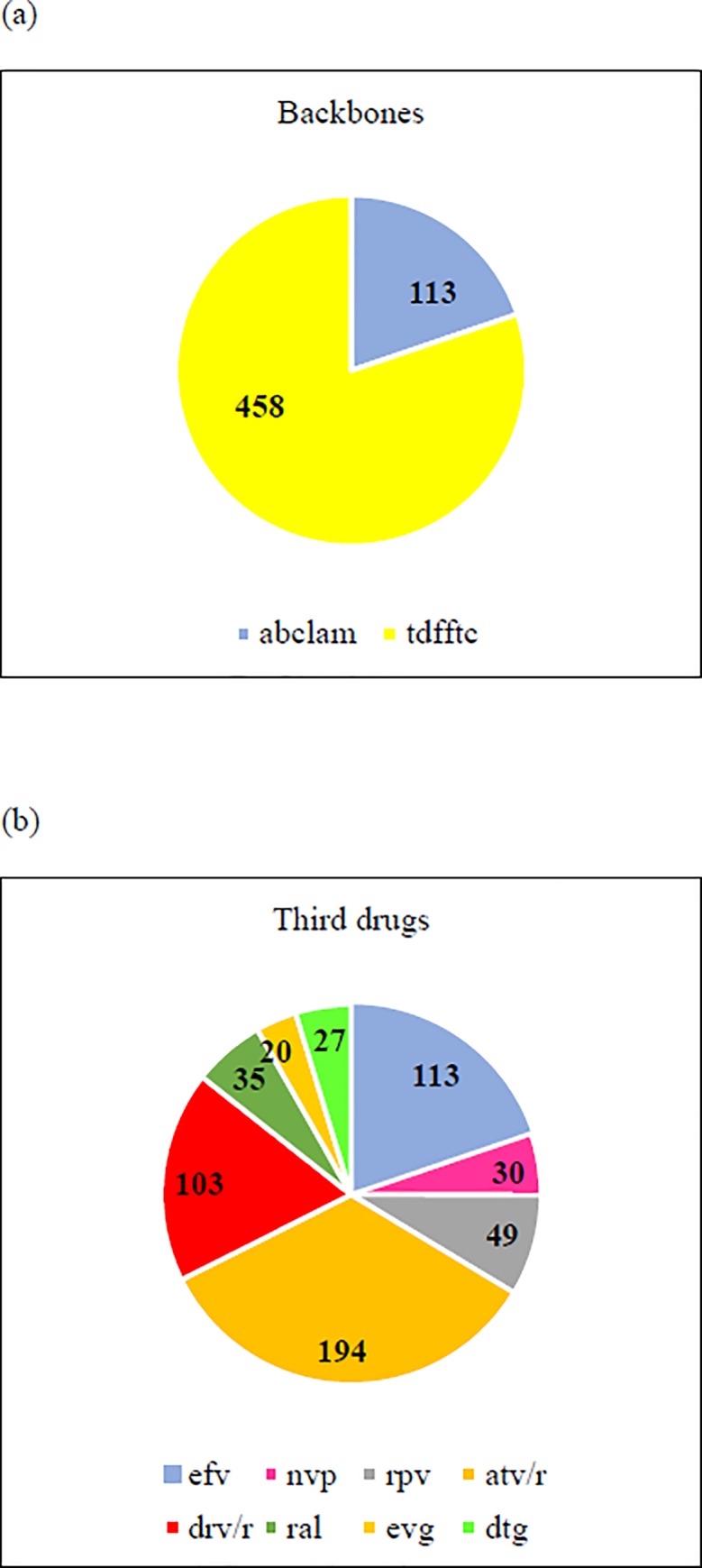
**Description of BBs (a) and third drugs (b) of the 571 patients included in the study**. Data are expressed as absolute numbers. (a) abclam: abacavir-lamivudine; tdfftc: tenofovir-emtricitabine (b) efv: efavirenz; nvp: nevirapine; rpv: rilpivirine; atv/r: atazanavir; drv/r: darunavir; ral: raltegravir; evg: elvitegravir; dtg: dolutegravir.

The data set comprised 577 HIV patients. Six subjects exhibited no plasma HIV viremia decrease after 2 months of therapy. The treating physician suspected that these patients were non-adherent, and they were not included in the statistical analysis. The baseline characteristics of the patients (120 subjects infected with X4 tropic virus according to an FPR 10%) are reported in Table 1. Sixty-one patients had an FPR value ≤ 5%, and their characteristics were comparable to those of the R5 subjects. Almost all the individuals (567, 99.3%) were white. The most frequently prescribed BB was tdfftc (80.2%), and overall, 82 patients (14.4%) were treated with INSTI. The descriptions of the BBs and third drugs prescribed to the 571 patients included in the study are provided in Fig 1. Pairwise correlation analyses of the main variables are reported in Table 2 and in Table 3. No correlation between BB and lrna6 was found (Pearson R-values 0.0001 for abclam and 0.0044 for tdfftc, no significant correlations at *p* <0.05). The percentages of subjects who achieved a plasma HIV RNA level lower than 50 copies/mL were comparable between the patients bearing X4 or R5 viruses with both FPRs (FPR = 5%: 68.3% vs. 75.4%; FPR = 10%: 71.4% vs. 75.5%).

**Table 1:** Main characteristics of 571 HIV-1 B subtype patients starting their first line cART.

	X4 types 120 patients 21%	R5 types 451 patients 79%	*p*
Male gender, n (%)	103 (85.8%)	358 (79.3)	0.1114
Age (years)^a^	41.5 (11.9)	40.4 (11.1)	0.3425
CD4+ cell count at T0 (cells/mm^3^)^a^	247 (259)	291 (205)	0.0493
HIV RNA at T0 (log_10_ copies/mL)^a^	5.74 (6.14)	5.72 (6.16)	0.9748
CD4+ cell count at T6 (cells/mm^3^)^a^	398 (256)	494 (265)	0.0005
HIV RNA at T6 (log_10_ copies/mL)^a^	3.33 (3.92)^b^	3.39 (4.31)^c^	0.9092
Patients with HIV RNA < 5 log_10_ copies/mL at T0, n (%)	50 (41.7)	190 (42.1%)	0.9275
Patients with HIV RNA 5–5.69 log_10_ copies/mL at T0, n (%)	40 (33.3)	175 (38.8%)	0.2722
Patients with HIV RNA > 5.69 log_10_ copies/mL at T0, n (%)	30 (25)	86 (19.1)	0.1516

^a^ mean and standard deviation

^b^34 patients with plasma HIV RNA > 50 copies/ml at T6

^c^110 patients with plasma HIV RNA > 50 copies/ml at T6; cART: combination antiretroviral treatment; T0: before cART; T6: 6^th^ months of cART

**Table 2:** Pairwise correlations between the main clinical variables: Analysis with FPR < 10. The correlations are Pearson R-values. Negative values indicate negative correlations.

	Age (years)	Female	x4_10	lcd4_0	lrna0	lrna6
**Age (years)**	1					
**Female**	-0.1027^a^	1				
**x4_10**	-0.0649	0.0436	1			
**lcd4_0**	-0.0432	-0.1535^a^	-0.1366^a^	1		
**lrna0**	-0.1112^a^	0.0880^a^	0.0243	-0.3527^a^	1	
**lrna6**	0.0483	0.0565	0.0528	-0.1754^a^	0.3235^a^	1

^a^Significant correlations (*p* < 0.05).

x4_10: co-receptor HIV tropism as X4; lcd4_0: log_10_-transformed CD4+ cell count at T0; lcd4_6: log_10_-transformed CD4+ cell count after 6 months of cART; lrna0: log_10_-transformed plasma HIV RNA at T0; lrna6: log_10_-transformed plasma HIV RNA after 6 months of cART

**Table 3:** Pairwise correlations between the main clinical variables: Analysis with FPR ≤ 5. The correlations are Pearson R-values. Negative values indicate negative correlations.

	Age (years)	Female	x4_5	lcd4_0	lrna0	lrna6
**Age (years)**	1					
**Female**	-0.1026^a^	1				
**x4_5**	0.1060^a^	-0.0539	1			
**lcd4_0**	-0.1504^a^	-0.0544	-0.1756^a^	1		
**lrna0**	0.0857^a^	-0.1041^a^	0.0677	-0.3554^a^	1	
**lrna6**	0.0517	0.0501	0.0819	-0.1766^a^	0.3247^a^	1

^a^Significant correlations (*p*  < 0.05).

x4_5: co-receptor HIV tropism as X4; lcd4_0: log_10_-transformed CD4+ cell count at T0; lcd4_6: log_10_-transformed CD4+ cell count after 6 months of cART; lrna0: log_10_-transformed plasma HIV RNA at T0; lrna6: log_10_-transformed plasma HIV RNA after 6 months of cART.

### Recursive partitioning

**Fig 2:**
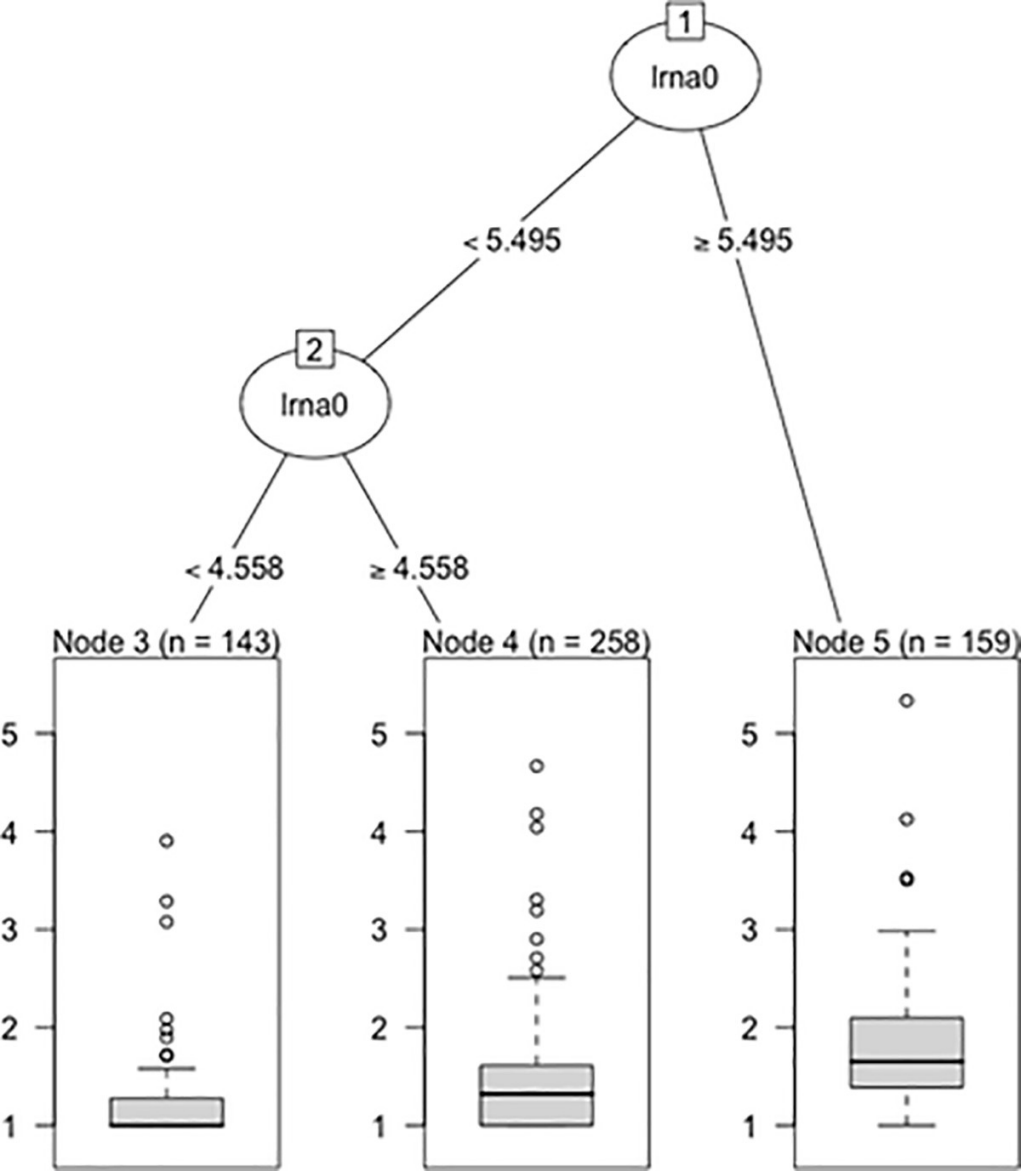
Regression tree by recursive partitioning: Description of lrna0 as predictor of lrna6. The first split was under the rule of lrna0 < or ≥ 5.495 (312,698 copies/mL), and the second was under the rule of lrna0 < or ≥ 4.558 (36,141 copies/mL). Three terminal nodes were produced. The distributions of lrna6 after partitioning are visually summarized. n: number of patients; lrna0: log_10_-transformed plasma HIV RNA at T0; lrna6: log_10_-transformed plasma HIV RNA after 6 months of cART.

After pruning of minor splits, the only important predictor left was lrna0, with 2 splits remaining. The first split was under the rule of lrna0 < or ≥ 5.495 log_10_ copies/mL, and the second was under the rule of lrna0 < or ≥ 4.558 log_10_ copies/mL. Three terminal nodes were produced: Node 3 with median lrna6 as 10 (IQR: 10, 19) copies/mL, Node 4 with median lrna6 as 21 (IQR: 10, 41) copies/mL, and Node 5 with median lrna6 as 45 (IQR: 24, 125) copies/mL (Fig 2).

#### Random forest regression

**Fig 3:**
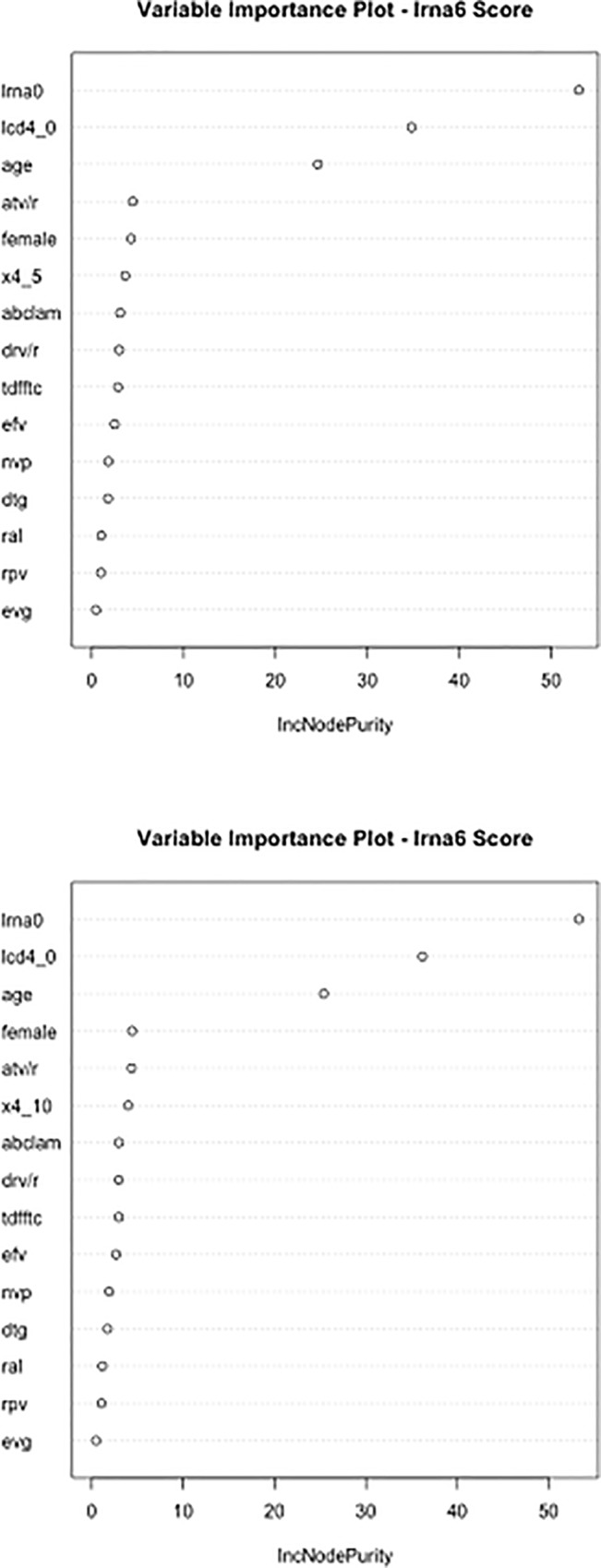
Importance table generated using lrna6 as outcome variable according to the increase of node purity induced by each predictive variable. Method: random forest. (a) FPR < 10% (b) FPR ≤ 5% lrna0: log_10_-transformed plasma HIV RNA at T0; lcd4_0: log_10_-transformed CD4+ cell count at T0; x4_10: co-receptor HIV tropism as X4 (FPR < 10%); x4_5: co-receptor HIV tropism as X4 (FPR ≤ 5%); lrna6: log_10_-transformed plasma HIV RNA after 6 months of cART; atv/r: atazanavir; drv/r: darunavir; tdfftc: tenofovir disoproxil fumarate/emtricitabine; abclam: abacavir/lamivudine; efv: efavirenz; dtg: dolutegravir; nvp: nevirapine; rpv: rilpivirine; ral: raltegravir; egv: elvitegravir.

The percentage variance of lrna6 explained was only 8.38 when FPR < 10 was applied and the percentage was 8.48 when FPR < 5 was chosen. An importance table was generated according to the increase of node purity shown by each predictive variable, and is depicted in Fig 3. The predictive importance of the antiviral agents appeared uniformly low, suggesting modest differences on late viral load by various drugs and various therapeutic schemes.

### Treatment effect

The analysis was performed only for efv, nvp, and atv/r, since for the other third drugs the probability of being treated yielded propensity scores < 1 × 10^−5^. Therefore treatment overlap assumption was violated. However, for efv, nvp, and atv/r the average treatment effect was not significant. The same analysis was performed with drug classes; whereas the estimation algorithm did not converge for INSTI, the others were not significant. In these cases, “not significance” attained the null hypothesis comparing an individual drug (or class) versus all other possible alternative drugs (or classes).

### Path analysis

#### Analysis with FPR < 10%

**Fig 4:**
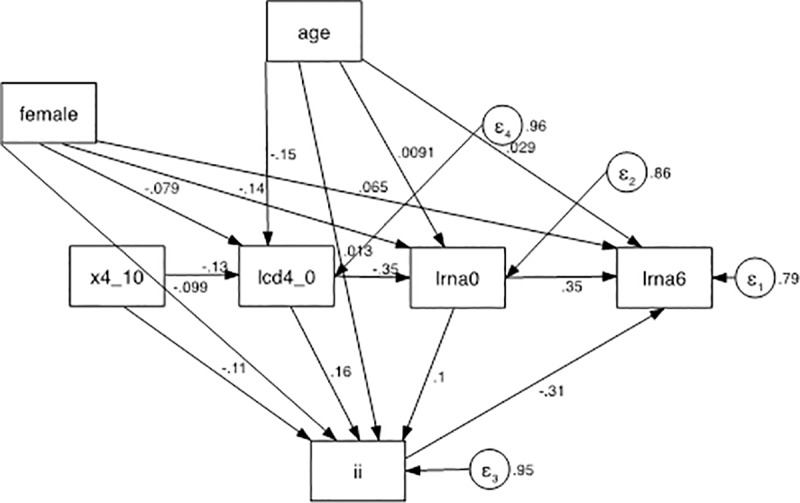
Path analysis model. There were 3 exogenous variables (age, female, and x4_10), 3 mediators (lcd4_0, lrna0, and ii), and a final dependent variable (outcome): lrna6. The treatment variable was ii, which mediated between (1) age, female, x4_10, lcd4_0, lrna0, and (2) lrna6. x4_10: co-receptor HIV tropism as X4; lcd4_0: log_10_ -transformed CD4+ cell count at T0; lcd4_6: log_10_-transformed CD4+ cell count after 6 months of ART; lrna0: log_10_-transformed plasma HIV RNA at T0; lrna6: log_10_-transformed plasma HIV RNA after 6 months of cART; INSTI: integrase inhibitors.

Baseline viremia exhibited often, but not always a significant positive effect on lrna6. When each third drug was examined singly, atv/r had a positive effect, i.e., it correlated with a higher residual viremia (*z* = 2.39, *p* = 0.017) compared to the average alternative third drug. The same approach was employed for drug classes. A significant direct positive effect of pi on lrna6 was found (higher residual viremia, *z* = 2.29, *p* = 0.022), and a significant negative effect of INSTI was also detected (*z* = -2.59, *p* = 0.010). The coefficients of the INSTI model are reported in Table 4 and the model is described in Fig 4.

**Table 4:** Path analysis aimed at estimating the treatment effect on lrna6 by INSTI in analysis with FPR < 10%. The 4 regression steps defining the path to the final outcome (lrna6) are reported in the first 4 sections of the table. Female, age, and x4_10 were exogenous variables, whereas lrna6, lrna0, lcd4_0, and therapy with INSTI were endogenous variables. The selection bias due to the choice of treatment was adjusted using INSTI as mediator variable, and all baseline information as explanatory set.

Standardized	Coef.	Std. Err.	*z*	*p*	95% Conf. Interval	
**lrna6**						
lrna0	0.3475996	0.0460461	7.55	0	0.2573509	0.4378483
INSTI	-0.3070178	0.118647	-2.59	0.01	-0.5395616	-0.074474
Female	0.0649282	0.0578053	1.12	0.261	-0.0483681	0.1782244
Age (years)	0.0293828	0.0450441	0.65	0.514	-0.058902	0.1176675
Intercept	0.4924667	0.3763257	1.31	0.191	-0.2451182	1.230052
**lrna0**						
lcd4_0	-0.3523528	0.0327359	-10.76	0	-0.416514	-0.2881916
Female	-0.1355738	0.0436358	-3.11	0.002	-0.2210984	-0.0500492
Age (years)	0.0090896	0.0419573	0.22	0.828	-0.0731452	0.0913244
Intercept	7.804966	0.3040944	25.67	0	7.208952	8.40098
**INSTI**						
lrna0	0.1029556	0.1038976	0.99	0.322	-0.10068	0.3065911
lcd4_0	0.1607515	0.0822961	1.95	0.051	-0.000546	0.3220489
Female	-0.0989271	0.0935878	-1.06	0.29	-0.2823558	0.0845015
Age (years)	0.0131043	0.0987351	0.13	0.894	-0.180413	0.2066216
x4_10	-0.113774	0.0795612	-1.43	0.153	-0.2697111	0.0421631
Intercept	-0.4668823	0.9372217	-0.5	0.618	-2.303803	1.370038
**lcd4_0**						
Female	-0.0794107	0.0414308	-1.92	0.055	-0.1606136	0.0017921
Age (years)	-0.1509164	0.0384372	-3.93	0	-0.2262518	-0.0755809
x4_10	-0.1323374	0.0444933	-2.97	0.003	-0.2195426	-0.0451322
Intercept	5.028884	0.2386328	21.07	0	4.561173	5.496596
var (e.lrna6)	0.7907716	0.0705402			0.6639269	0.9418502
var (e.lrna0)	0.8616431	0.0249203			0.8141589	0.9118968
var (e.ii)	0.9460575	0.0448505			0.8621125	1.038176
var (e.lcd4_0)	0.9550003	0.0168778			0.9224868	0.9886598

Discr. test of model vs. saturated: chi2(3) = 0.56, Prob > chi2 = 0.9052

x4_10: co-receptor HIV tropism as X4 with FPR ≤ 10%; lcd4_0: log_10_-transformed CD4+ cell count at T0; lcd4_6: log_10_-transformed CD4+ cell count after 6 months of cART; lrna0: log_10_-transformed plasma HIV RNA at T0; lrna6: log_10_-transformed plasma HIV RNA after 6 months of cART; INSTI: integrase inhibitors

From the model, the mean residual viremia was 40 copies/mL without INSTI and 3 copies/mL with INSTI.

#### Analysis with FPR ≤ 5%

A significant direct effect on lrna6 was detected for atv, favoring an higher mean outcome viremia (*z* = 2.57, *p* = 0.010), and for ral, predicting lower final levels of viremia (*z* = 2.57, *p* = 0.010).

With evg, lrna0 appeared to lose its regular, significant predictive effect on lrna6.

**Fig 5:**
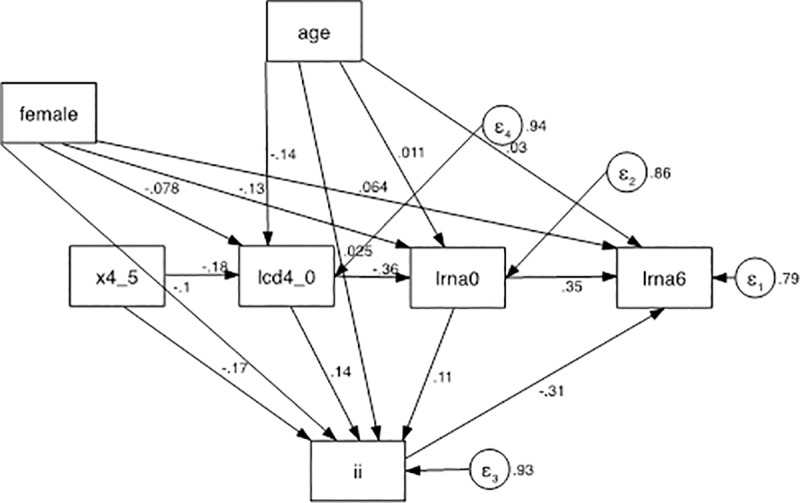
Path analysis model. There were 3 exogenous variables (age, female, and x4_5), 3 mediators (lcd4_0, lrna0, and ii), and a final dependent variable (outcome): lrna6. The treatment variable was ii, which mediated between (1) age, female, x4_10, lcd4_0, lrna0, and (2) lrna6. x4_5: co-receptor HIV tropism as X4; lcd4_0: log_10_-transformed CD4+ cell count at T0; lcd4_6: log_10_-transformed CD4+ cell count after 6 months of cART; lrna0: log_10_-transformed plasma HIV RNA at T0; lrna6: log_10_-transformed plasma HIV RNA after 6 months of cART; INSTI integrase inhibitors.

By estimating the effects of the third drug classes, a significant direct positive effect of pi on lrna6 was found (higher residual viremia, *z* = 2.28, *p* = 0.022), and a significant negative effect of INSTI was also detected (lower residual viremia, *z* = -3.00, *p* = 0.003). The coefficients of the INSTI model are reported in Table 5 The same model is depicted in Fig 5.

**Table 5:** Path analysis aimed at estimating the treatment effect on lrna6 by INSTI in analysis with FPR ≤ 5%. The 4 regression steps defining the path to the final outcome (lrna6) are reported in the first 4 sections of the table. Female, age, and x4_5 were exogenous variables, whereas lrna6, lrna0, lcd4_0, and therapy with INSTI were endogenous variables. The selection bias due to the choice of treatment was adjusted using INSTI as mediator variable, and all baseline information as explanatory set.

Standardized	Coef.	Std. Err.	*z*	*p*	95% Conf. Interval	
**lrna6**						
lrna0	0.3472	0.0465	7.47	0.000	0.2561	0.4383
INSTI	-0.3108	0.1036	-3.00	0.003	-0.5139	-0.1077
Female	0.0637	0.0565	1.13	0.260	-0.0471	0.1745
Age	0.0299	0.0450	0.66	0.507	-0.0584	0.1181
Intercept	0.5082	0.3640	1.40	0.163	-0.2051	1.2216
**lrna0**						
lcd4_0	-0.3567	0.0330	-10.82	0.000	-0.4213	-0.2921
Female	-0.1348	0.0435	-3.10	0.002	-0.2199	-0.0496
Age	0.0113	0.0419	0.27	0.786	-0.0707	0.0934
Intercept	7.7775	0.3047	25.53	0.000	7.1803	8.3746
**INSTI**						
lrna0	0.1064	0.1028	1.03	0.301	-0.0951	0.3078
lcd4_0	0.1420	0.0831	1.71	0.088	-0.0209	0.3050
Female	-0.1027	0.0911	-1.13	0.260	-0.2812	0.0759
Age	0.0252	0.0997	0.25	0.800	-0.1702	0.2206
x4_5	-0.1699	0.0819	-2.07	0.038	-0.3305	-0.0094
Intercept	-0.4353	0.9335	-0.47	0.641	-2.2649	1.3943
**lcd4_0**						
Female	-0.0783	0.0409	-1.91	0.056	-0.1586	0.0019
Age	-0.1385	0.0380	-3.64	0.000	-0.2131	-0.0640
x4_5	-0.1750	0.0438	-4.00	0.000	-0.2608	-0.0892
Intercept	4.9821	0.2383	20.91	0.000	4.5151	5.4490
var(e.lrna6)	0.7897	0.0638			0.6740	0.9251
var(e.lrna0)	0.8583	0.0253			0.8102	0.9093
var(e.ii)	0.9316	0.0518			0.8354	1.0390
var(e.lcd4_0)	0.9422	0.0190			0.9057	0.9801

x4_5: co-receptor HIV tropism as X4 with FPR ≤ 5%; lcd4_0: log_10_-transformed CD4+ cell count at T0; lcd4_6: log_10_-transformed CD4+ cell count after 6 months of cART; lrna0: log_10_-transformed plasma HIV RNA at T0; lrna6: log_10_-transformed plasma HIV RNA after 6 months of cART; INSTI: integrase inhibitors.

The model also predicted a mean residual viremia of 40 copies/mL without INSTI and 3 copies/mL with INSTI.

## Discussion

The achievement of viral suppression preserves immune function, reduces the risk of HIV transmission, and increases life expectancy [15,16]. Lee et al. [17] reported a gradual increase in the standardized hazard ratio of estimates of 10-year all-cause mortality with increasing viral load that was discernible at 130 copies/mL with respect to patients with a plasma HIV RNA value <20 copies/mL after 6 months of cART in a clinical cohort of 7944 subjects with a median pretreatment CD4+ cell count of 349 cells/mm^3^, which is higher than that reported in our study.

It is difficult to measure treatment effect in an observational setting. Here, the choice of treatment was assigned outside of a randomization process, which only allows for the assurance of the equivalence of the possible confounder variables superimposed on the various treatment conditions. To classify the predictive role of each variable, 2 related estimators were employed, i.e., recursive partitioning and random forest. These estimators unveiled the modest overall predictive power of all of the covariates (~8% of the lrna6 variance) and the minimal predictive performances of the individual cART agents. The overwhelming weight of the baseline viremia confirmed the results of our previous study [5].

Next, quantitative evaluation of the antiviral effect was sought with the treatment effect estimator available with Stata. This approach was applicable only to efv, nvp, and atv/r drugs and appeared devoid of any significant effect in explaining the variations of the antiviral effects. The failure in the attempts to evaluate rpv, drv, ral, dth, and evg were clearly due to the strong ties that existed between the covariates.

Ultimately, path analysis appeared to offer a possibility of adjusting for these ties. A significant direct effect of the baseline viremia and an indirect positive effect of carrying an X4 virus on lrna6 were detected with both FPRs of 5% and 10%. These results agreed with the results of our previous study of a population of naïve patients whose cART did not include INSTI [5]. The threshold of 10% was used as it is recommended by the European guidelines in clinical settings, and the analysis based on the more selective cut-off of 5% was added to confirm the role of having an X4 tropic virus infection even if it was in an expected lower number of subjects [13]. Patients with an X4 virus infection, if left untreated, exhibit faster disease progression and decreases in CD4+ cell counts [18,19]. Despite the clear benefits of starting cART, some HIV-naïve patients are still present in high-income countries for several reasons, including the belief that the disease can be controlled without medication, and thus medication is the last resort [20,21]. Furthermore, hypertension, renal impairment, and metabolic and bone disorders have higher prevalences in X4 subjects with ongoing successful cART with FPR thresholds of both of 5% and 5–10% (adjusted hazard risks 1.89 and 2.02, respectively, compared with R5 viruses with FPRs >60%) [22]. Taken together, these two aspects suggest that pretreatment determination of tropism should be performed to motivate patients and to tailor surveillance strategies so that non AIDS events may be identified early.

The path analysis detected a significant positive effect of pi class and a significant negative effect of INSTI class on lrna6. These results favor a greater comparative antiviral effect of INSTI. Interestingly, in almost all the patients, lrna0 had no more significant effect on lrna6 in the patients treated with INSTI. The effect of RAL on lrna6 was significant when the analysis was performed with FPR ≤ 5% and not when FPR 10%. This last result accords with those of Rusconi et al. [23] who reported on a cohort of selected triple-class-experienced subjects who were failing their current treatment and were treated with a RAL-containing cART in a clinical practice setting, and with those of Raffi et al. [24] that were obtained in a randomized controlled trial that enrolled naïve subjects who were treated with NRTI BBs and dtg or ral.

Moreover, we demonstrated that the subjects treated with INSTI as the third drug achieved a mean predicted residual viremia of 3 copies/mL after 6 months of therapy, which was lower than the value obtained in patients treated with other third drugs (40 copies/mL). Low ranges of plasma HIV viremia correlate with lower levels of systemic inflammatory markers. Bastard et al. [25] described a significant positive increase in serum IL-6 levels with a cut-off of 31 copies/mL in HIV patients treated with all third-class drugs, and Baroncelli et al. [26] reported a reduced level of lipopolysaccharide in subjects with a suppression of viral replication as defined as < 2.5 copies/mL. Negative correlations of log_10_DNA, baseline log_10_RNA and the previously identified residual viremia threshold were demonstrated by Parisi et al. [27] in a cohort of cART-naive subjects not including INSTI, which suggests that INSTI as a first therapeutic approach could influence the long-term HIV reservoir burden [28,29].

The strengths of this study are the statistical approach, the inclusion of subjects who were treated with all available cART regimens prescribed as first-line therapies, and the comparable clinical and demographic characteristics of the X4 and R5 subjects included. No differences other than a lower CD4+ cell number in the X4 patients with respect to the R5 subjects (247 cells/mm^3^ versus 291 cells/mm^3^) were found. These were expected results [30]. Nonetheless, the CD4+ cell value was comparable to that reported in the study by Bouteloup et al. [31], which was conducted on 28,992 naïve patients living in 35 European countries (249 cells/mm^3^). The values of lrna0, both as continuous data and as categorized into 3 classes, were not significantly different between the X4 and R5 subjects. Only subjects with HIV subtype B were included because of the insufficient sensitivity of the methods in non-B HIV strains [32]; this is the main limit of this study.

Path analysis appears to be a useful resource for treatment effect evaluations in observational clinical studies, particularly in the field of HIV therapy in which more than one therapeutic option is available, and the integrated study of baseline variables, including tropism, could help in the tailoring of cost-effective cART choices.
